# Erlotinib-based doublet targeted therapy versus erlotinib alone in previously treated advanced non-small-cell lung cancer: a meta-analysis from 24 randomized controlled trials

**DOI:** 10.18632/oncotarget.18319

**Published:** 2017-05-31

**Authors:** Jian-Wei Gao, Ping Zhan, Xiang-Yu Qiu, Jia-Jia Jin, Tang-Feng Lv, Yong Song

**Affiliations:** ^1^ Department of Respiratory Medicine, Jinling Hospital, Nanjing University School of Medicine, Nanjing, China; ^2^ The Research Institute of General Surgery, Jinling Hospital, Nanjing University School of Medicine, Nanjing, China

**Keywords:** erlotinib, targeted therapy, advanced non-small cell lung cancer, meta-analysis

## Abstract

**Background:**

To assess the efficacy profile of erlotinib-based doublet targeted therapy compared with erlotinib monotherapy for previously treated patients with advanced NSCLC, a meta-analysis was performed.

**Patients and methods:**

We rigorously searched PubMed, Embase, Cochrane and meeting proceedings. Phase II/III randomized trials reporting on the efficacy of erlotinib-doublet therapy *versus* single-agent therapy were selected. We estimated the HR for OS, PFS and the RR for ORR, DCR, 1-year SR. Phases of trials, targeted signaling pathways, *EGFR*-status and *KRAS*- status were included in subset analysis.

**Results:**

24 studies involving 6,196 patients were eligible. In general, the combination targeted therapy significantly improved PFS, ORR and DCR. There was also a trend showing improved OS and 1-year SR in doublets group, though it was not statistically significant. Subgroup analysis suggested PFS improvement in *EGFR* wild-type, *KRAS* mutant, *KRAS* wild-type populations. Moreover, patients treated with anti-angiogenesis or anti-MET targeted agent revealed a significant benefit in PFS.

**Conclusion:**

In patients with advanced NSCLC, erlotinib-doublets target therapy (specially combination with anti-angiogenesis and anti-MET targeted agents) was associated with a statistically significantly longer PFS, greater ORR and DCR, but the combination did not improve OS and 1-year SR compared with erlotinib alone.

## INTRODUCTION

Based on the most recent WHO estimate, lung cancer is a leading cause of cancer-related mortality with approximately 1·59 million deaths worldwide in 2012. [1] In China, lung cancer is estimated to account for 21.6% of all cancer deaths in 2015. [2]

In patients with advanced non-small-cell lung cancer (NSCLC), platinum-doublet chemotherapy is standard treatment in the first-line setting; however, most patients ultimately progress and survived for less than 1 year. [3] Discovery and subsequent targeting of the epidermal growth factor receptor (EGFR) pathway has imparted clinical benefit and ushered in a new era of targeted therapeutic agents for patients with NSCLC. Several guidelines recommend EGFR tyrosine kinase inhibitors (TKIs), such as erlotinib, as an option of second- or third-line treatments for advanced NSCLC, independent of the EGFR mutational status. [4] Nonetheless, prognosis remains poor; the median progression-free survival (PFS) for patients treated with erlotinib monotherapy, regardless of EFGR mutation status, is still only around 2.2 months after failure with platinum salts and overall survival was 6.7 months according to a placebo-controlled trial conducted by Shepherd et al. [5]

Multiple signaling pathways recognized to play key roles in homeostatic processes have been identified as key drivers of oncogenesis through genetic and epigenetic aberrations, including ErbB receptor tyrosine kinases, anaplastic lymphoma kinase (ALK), insulin-like growth factor-1 receptor (IGF-1R), hepatocyte growth factor (HGF)-mesenchymal-epithelial transition factor (MET) axis, to name a few. [6] Given the heterogeneity of NSCLC and potential crosstalk between signaling pathways implicated in tumor growth, angiogenesis and metastasis, combining targeted agents could improve the efficacy over single-target agents,, which could also be necessary to reverse resistance to EGFR inhibitor therapy. [6–8]

Several trials have been conducted to evaluate benefits of combining targeted agent with erlotinib compared with erlotinib alone, especially the agents targeting angiogenesis, MET, IGF-1R and ErbB3 signaling. However, the results from these trials were controversial and some were of small sample size. This meta-analysis intended to pool and analyze all relevant randomized phase II/III trials, which provided a more precise assessment of efficacy of erlotinib-doublet targeted therapy compared with monotherapy in subsequent lines after previously treated with standard chemotherapy. Predefined subgroup analysis was conducted to identify the potential appropriate patient population to benefit from such combined therapy.

## RESULTS

### Literature search

**Figure 1:**
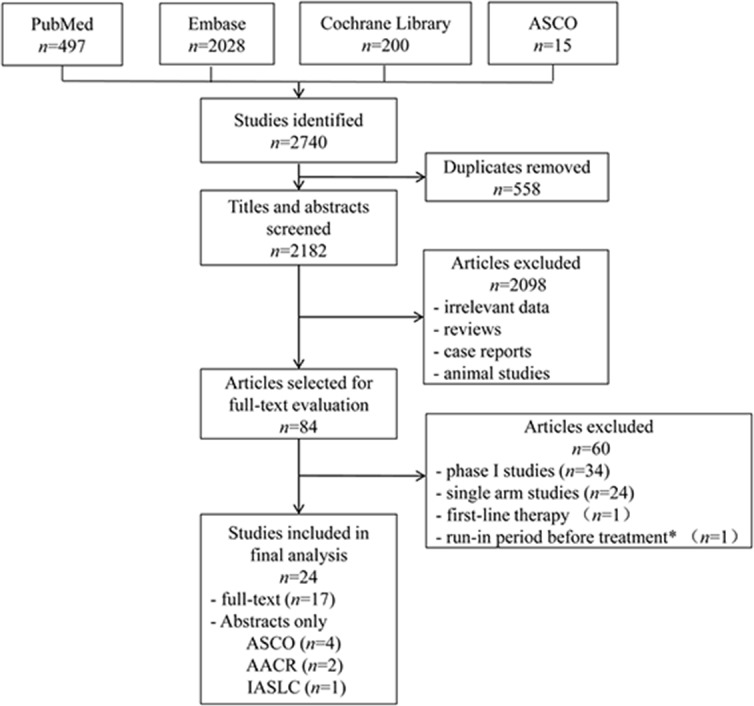
Flowchart of the process for selecting relevant articles ASCO, American Society of Clinical Oncology; AACR, American Association for Cancer Research; IASLC, International Association for the Study of Lung Cancer. *Patients entered an open-label run-in period where they received single-agent apricoxib (400 mg/day) for 5 consecutive days.

We identified 2,740 initial article candidates, and 24 articles involving 6,196 patients met the inclusion criteria after rigorously identification (Figure 1). 2,656 articles were excluded based on the title and abstract for the following reasons: duplicates, irrelevant data, reviews, case reports, animal studies. The rest 84 articles were retrieved for full-text review, from which 60 were removed: 34 phase I trials, 24 single-arm phase II trials, 1 focusing on first-line therapy, 1 involving in a run-in period where patients received the study drug. The remaining 17 trials [9–25] with full-text and 7 additional conference abstracts [26–32] were included in the final analysis.

### Study characteristics

The detailed characteristics of eligible studies are summarized in Table 1 and Table 2. Of the 24 randomized trails, the primary end point was PFS in twelve [11, 16–18, 20, 23, 25, 26, 28, 30–32], OS in six [12, 14, 21, 22, 24, 29], ORR in two [9, 10, 13, 27], ORR plus PFS (coprimary end points) in one [10], 12-weeks PFS rate in one [13], 4-momth PFS rate in one [15] and DCR at 3 months in one [19]. Six [12, 14, 21, 22, 24, 29] of the included studies were phase III RCTs and the remaining were phase II RCTs. 14 trials [10–15, 17, 18, 22–24, 26, 29, 30] employed erlotinib plus placebo as the control arm, while the remaining 10 treated control subjects with single-agent erlotinib. 8 studies tested targeted therapies in molecularly enriched populations in accordance with *EGFR* status (immunocytochemistry positive [16]; wild-type [24, 31, 32]), *KRAS* status (wild-type) [25], expression of MET (immunocytochemistry 2+/3+) [29] and histological type (non-adenocarcinoma [21]; non-squamous cell carcinoma [22, 24, 32]). Due to two three-arm trials, each of which consisted of two comparisons with a shared control, there were four comparisons for OS and PFS from these two studies.[13, 30] One article investigated two parallel randomized phase II trials, yet only one trial was of interest in our review.[25] All of the included studies provided sufficient data about OS, PFS and ORR except two [25, 28] without value of HR or 95% CI for survival data and one [30] without ORR. Data for DCR and 1-year SR were available in 16 [9–14, 16, 19–25, 31, 32] and 17 [9–18, 21–24, 29, 31, 32] trials, respectively.

**Table 1:** Study characteristics of the randomized trials Included in the meta-analysis

Study	Year	Phase	Group	Targeted signaling	Selected populations	N	Age, years	Female, %	Smoking, %	Histology, AC/SCC, %	ECOG PS,0/1,%	Stage, IIIB/IV, %	prior chemotherapy regimens, 1/≥2,%
Lynch[9]	2009	II	Erl + bortezomib	proteasome inhibitor	unselected	25	62	56	84	60/28	29/67	16/84	4(0)/76/20
			Erl			25	64	48	80	56/28	28/72	12/88	12(0)/84/4
Herbst[12]	2011	III	Erl + bevacizumab	anti-VEGF monoclonal antibody	unselected	319	65	46	89	76/3	41/52	NA	NA
			Erl + placebo			317	65	46	90	74/5	38/56	NA	NA
Ramalingam[13]	2011	II	Erl + R1507 (9 mg/kg/wk)	anti-IGF-1R monoclonal antibody	unselected	57	63	32	86	46/26	NA	19/81	77/23
			Erl + R1507 (16 mg/kg/3wks)			57	62	33	91	44/28	NA	12/88	68/32
			Erl + placebo			57	62	35	84	63/21	NA	19/81	75/25
Sequist[11]	2011	II	Erl + tivantinib	MET inhibitor	unselected	84	64	39	80	56/31	27/71	10/91	60/40
			Erl + placebo			83	62	41	78	65/29	20/80	13/87	61/39
Spigel[10]	2011	II	Erl + sorafenib	TKI against VEGFR2/3, PDGFRB	unselected	111	65	44	83	NA/33	29/56	NA	66/34
			Erl + placebo			55	65	53	85	NA/31	29/51	NA	51/49
Scagliotti[14]	2012	III	Erl + sunitinib	TKI against VEGFR, PDGFRA/B	unselected	480	61	38	80	57/28	38/61	9/91	71/29
			Erl + placebo			480	61	41	81	54/28	37/63	7/93	71/29
Spigel/IASLC[26]	2012	II	Erl + pazopanib	TKI against VEGFR, PDGFRA/B	unselected	134	66	47	96	NA/22	NA	NA	61/39
			Erl + placebo			67	67	42	91	NA/26	NA	NA	65/35
Witta[15]	2012	II	Erl + entinostat	HDACi	unselected	67	66	42	84	58/27	43/45	NA	NA
			Erl + placebo			65	67	34	83	43/32	34/52	NA	NA
Belani[16]	2013	II	Erl + PF-3512676	TLR9 agonist	EGFR-IHC positive	21	63	57	90	62/33	90(0/1)	NA	57/43
			Erl			22	64	41	86	64/9	91(0/1)	NA	86/14
Garon/AACR[27]	2013	II	Erl + fulvestrant	Estrogen antagonist	unselected	72	NA	NA	NA	NA	NA	NA	NA
			Erl			34	NA	NA	NA	NA	NA	NA	NA
Groen[18]	2013	II	Erl + sunitinib	TKI against VEGFR, PDGFRA/B	unselected	65	59	40	88	55/23	32/66	2/97	60/37
			Erl + placebo			67	61	33	85	46/28	31/67	0/100	69/31
Spigel[17]	2013	II	Erl + onartuzumab	anti-MET monoclonal antibody	unselected	69	64	42	86	58/29	32/62	NA	NA
			Erl + placebo			68	63	38	88	61/29	31/66	NA	NA
Besse[19]	2014	II	Erl + everolimus	mTOR inhibitor	unselected	66	60	46	80	70/15	NA	12/78	77/23
			Erl			67	61	50	81	69/15	NA	19/63	61/37
Moran[20]	2014	II	Erl + dalotuzumab	anti-IGF-1R monoclonal antibody	unselected	37	62	27	89	38/30	30/65	11/89	NA
			Erl			38	59	26	71	40/16	34/63	24/76	NA
Oton/AACR[28]	2014	II	Erl + Efatutazone	PPARγ agonist	unselected	45	60	24	69	NA	NA	NA	NA
			Erl			45	61	44	54	NA	NA	NA	NA
Pawel/ASCO[30]	2014	II	Erl + patritumab (18 mg/kg/3wks)	anti-ErbB3 monoclonal antibody	unselected	70	62	46	86	66/27	47/53	NA	71/29
			Erl + patritumab (9 mg/kg/3wks)			71	65	32	85	62/32	42/58	NA	68/32
			Erl + placebo			71	60	39	93	60/30	35/65	NA	66/34
Sequist/ASCO[31]	2014	II	Erl + MM-121	anti-ErbB3 monoclonal antibody	WT-EGFR	85	65	41	84	NA	NA	NA	32/68
			Erl			44	64	39	71	NA	NA	NA	39/61
Spigel/ASCO[29]	2014	III	Erl + onartuzumab	anti-MET monoclonal antibody	MET-IHC 2+/3+	250	62	44	NA	NA/16	37/61	NA	NA
			Erl + placebo			249	63	44	NA	NA/12	31/68	NA	NA
Neal /ASCO[32]	2015	II	Erl + cabozantinib	TKI against MET,VEGFR2	non-SCC, WT-EGFR	36	63	NA	83	NA	25/64	NA	NA
			Erl			38	66	NA	87	NA	24/63	NA	NA
Reckamp[23]	2015	II	Erl + celecoxib	COX-2 inhibitor	unselected	54	64	52	63	59/11	48/52	11/89	11(0)/50/39
			Erl + placebo			53	65	55	62	60/9	49/51	8/92	13(0)/51/36
Scagliotti-fig[21]	2015	III	Erl + figitumumab	anti-IGF-1R monoclonal antibody	non-AC	293	62	22	94	0/90	81(0/1)	21/78	NA
			Erl			290	62	22	91	0/91	82(0/1)	19/81	NA
Scagliotti-tiv[22]	2015	III	Erl + tivantinib	MET inhibitor	non-SCC	526	62	41	81	91/0	32/68	4/95	66/34
			Erl + placebo			522	61	41	81	95/0	32/68	3/96	67/33
Yoshioka[24]	2015	III	Erl + tivantinib	MET inhibitor	non-SCC, WT-EGFR	154	63	29	73	NA	43/57	4/96	60/40
			Erl + placebo			153	63	33	75	NA	33/67	6/94	59/41
Carter[25]	2016	II	Erl + selumetinib	MEK kinase inhibitor	WT-KRAS	19	84	47	64	79/21	10/37	NA	42/58
			Erl			19	68	32	64	79/21	10/58	NA	52/48

ECOG PS, Eastern Cooperative Oncology Group Performance Status; Erl, erlotinib; VEGF, vascular endothelial growth factor; VEGFR, vascular endothelial growth factor receptor; IGF-1R, insulin-like growth factor-1 receptor; HDACi, selective histone deacetylase inhibitor; MET, mesenchymal-epithelial transition factor; TKI, tyrosine kinase inhibitor; PDGFR, platelet-derived growth factor receptor; TLR9, Toll-like receptor 9; mTOR, mammalian target of rapamycin; PPARγ, peroxisome proliferative activated receptor γ; COX-2, cyclo-oxygen-ase-2; MEK, AC, adenocarcinoma; SCC, squamous cell carcinoma; EGFR, epidermal growth factor receptor; IHC, immunohistochemistry; WT, wild-type; NA, not applicable;

**Table 2:** Study outcomes of the randomized trials included in the meta-analysis

Study	Group	Primary endpoint	ORR, %	DCR, %	1-year SR, %	OS, mo	PFS, mo	WT-EGFR			Mut-EGFR		
								N	OS, mo	PFS, mo	N	OS, mo	PFS, mo
Lynch[9]	Erl + bortezomib	ORR	8.0	40.0	30	8.5	1.3	12	NA	NA	2	NA	NA
	Erl		16.0	52.0	40	7.3	2.7	11	NA	NA	4	NA	NA
Herbst[12]	Erl + bevacizumab	OS	11.9	42.6	42.1	9.3	3.4	173	8.1	NA	12	NA	NA
	Erl + placebo		6.0	32.8	40.7	9.2	1.7	152	9.1	NA	18	20.2	NA
Ramalingam[13]	Erl + R1507(9 mg/kg/wk)	12-wk PFS rate	8.8	49.1	30.1	8.1	1.9	NA	NA	NA	2	NA	NA
	Erl + R1507(16 mg/kg/3wks)		7.0	56.1	50.6	12.1	2.7	NA	NA	NA	1	NA	NA
	Erl + placebo		8.8	49.1	33.1	8.1	1.5	NA	NA	NA	3	NA	NA
Sequist[11]	Erl + tivantinib	PFS	8.3	57.1	28.3	8.5	3.8	51	NA	3.2	6	NA	5.6
	Erl + placebo		6.0	47.0	32.4	6.9	2.3	48	NA	1.9	11	NA	4.9
Spigel[26]	Erl + sorafenib	ORR/PFS	8.1	54.1	32.7	7.6	3.4	43	8.1	3.4	2	NA	NA
	Erl + placebo		10.9	38.2	40.3	7.2	1.9	24	4.5	1.8	3	NA	9.2
Scagliotti[14]	Erl + sunitinib	OS	10.6	42.9	40	9.0	3.6	NA	NA	NA	NA	NA	NA
	Erl + placebo		6.9	35.0	37	8.5	2.0	NA	NA	NA	NA	NA	NA
Spigel/IASLC[26]	Erl + pazopanib	PFS	9.0	NA	NA	6.8	2.6	NA	NA	NA	NA	NA	NA
	Erl + placebo		4.5	NA	NA	6.7	1.8	NA	NA	NA	NA	NA	NA
Witta[15]	Erl + entinostat	4-month PFS rate	3.0	NA	39.2	8.9	2.0	33	NA	NA	3	NA	NA
	Erl + placebo		9.2	NA	28.9	6.7	1.9	43	NA	NA	3	NA	NA
Belani[16]	Erl + PF-3512676	PFS	9.5	19.1	34.3	6.4	1.6	9	NA	NA	4	NA	1.6
	Erl		4.6	18.2	15.3	4.7	1.7	14	NA	NA	2	NA	1.7
Garon/AACR[27]	Erl + fulvestrant	ORR	23.6	NA	NA	9.4	1.9	38	7.4	2.0	14	NA	NA
	Erl		14.7	NA	NA	5.7	1.8	14	5.9	1.6	7	NA	NA
Groen[18]	Erl + sunitinib	PFS	4.6	NA	32	8.2	2.8	21	NA	NA	4	NA	NA
	Erl + placebo		3.0	NA	42	7.6	2.0	19	NA	NA	1	NA	NA
Spigel[17]	Erl + onartuzumab	PFS	5.8	NA	36	8.9	2.2	49	8.5	NA	10	NA	NA
	Erl + placebo		4.4	NA	30.7	7.4	2.6	50	7.4	NA	9	NA	NA
Besse[19]	Erl + everolimus	DCR at 3 months	12.1	57.6	NA	9.1	2.9	NA	NA	NA	NA	NA	NA
	Erl		10.5	38.8	NA	9.7	2.0	NA	NA	NA	NA	NA	NA
Moran[20]	Erl + dalotuzumab	PFS	2.7	59.5	NA	6.6	2.5	NA	NA	NA	NA	NA	NA
	Erl		7.9	63.2	NA	10.2	1.6	NA	NA	NA	NA	NA	NA
Oton/AACR[28]	Erl + Efatutazone	PFS	20.5	NA	NA	7.6	4.1	NA	NA	NA	NA	NA	NA
	Erl		20.0	NA	NA	11.4	2.8	NA	NA	NA	NA	NA	NA
Pawel/ASCO[30]	Erl + patritumab(18 mg/kg/3wks)	PFS	NA	NA	NA	NA	1.4	17	NA	NA	0	NA	NA
	Erl + patritumab(9 mg/kg/3wks)		NA	NA	NA	NA	2.5	21	NA	NA	2	NA	NA
	Erl + placebo		NA	NA	NA	NA	1.6	23	NA	NA	2	NA	NA
Sequist/ASCO[31]	Erl + MM-121	PFS	4.7	40.0	27.1	6.3	1.9	85	6.3	1.9	0	NA	NA
	Erl		4.6	29.6	24.8	9.3	1.8	44	9.3	1.8	0	NA	NA
Spigel/ASCO[29]	Erl + onartuzumab	OS	8.4	NA	27.3	6.8	2.7	222	6.4	2.6	28	12.6	NA
	Erl + placebo		9.6	NA	33.0	9.1	2.6	220	7.8	1.5	29	NA	8.5
Neal/ASCO[32]	Erl + cabozantinib	PFS	5.6	36.1	58.8	13.3	4.7	36	13.3	4.7	0	NA	NA
	Erl		2.6	15.8	17.6	4.1	1.9	38	4.1	1.9	0	NA	NA
Reckamp[23]	Erl + celecoxib	PFS	22.2	63.0	53.7	12.9	5.4	31	9.8	3.2	12	NA	9.2
	Erl + placebo		32.1	56.6	60.4	14	3.5	27	10.9	1.8	14	NA	9.2
Scagliotti-fig[21]	Erl + figitumumab	OS	5.5	44.0	24.5	5.7	2.1	NA	NA	NA	NA	NA	NA
	Erl		3.8	48.6	24.9	6.2	2.6	NA	NA	NA	NA	NA	NA
Scagliotti-tiv[22]	Erl + tivantinib	OS	10.3	45.8	35.9	8.5	3.6	469	7.2	2.7	56	NA	NA
	Erl + placebo		6.5	32.0	34.1	7.8	1.9	468	7.1	1.9	53	NA	NA
Yoshioka[24]	Erl + tivantinib	OS	8.4	39.0	54.4	12.7	2.9	154	12.7	2.9	0	NA	NA
	Erl + placebo		6.5	32.0	47.6	11.1	2.0	153	11.1	2.0	0	NA	NA
Carter[25]	Erl + selumetinib	PFS	12.0	35.0	NA	12.9	2.1	18	NA	NA	1	NA	NA
	Erl		5.0	47.0	NA	6.3	2.4	18	NA	NA	1	NA	NA

ORR, objective response rate; DCR, disease control rate; SR, survival rate; OS, overall survival; PFS, progression-free survival; WT, wild-type; Mut, mutant; mo, months; Erl, erlotinib; wk, weeks; NA, not applicable; EGFR, epidermal growth factor receptor; ASCO, American Society of Clinical Oncology; AACR, American Association for Cancer Research; IASLC, International Association for the Study of Lung Cancer

### Risk of bias

All the included trials reported “randomization” with 75% and 54% studies providing the conduction details of random sequence generation and allocation concealment, respectively. 10 RCTs were marked with “open-label” and the performance bias was assessed as “high risk”. For other key domains, no high risk of bias was detected. Full details of the assessment are in Supplementary Table 1.

### Efficacy outcomes

**Figure 2:**
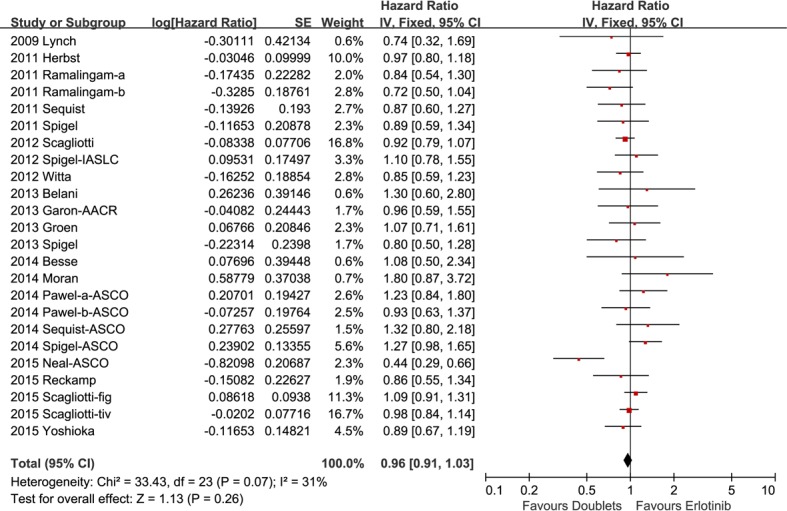
Forest plots for overall survival

The median OS were 5.7 to 13.3 months in the combination arm versus 4.1 to 14 months in the control arm. Pooled HR for OS estimated from 22 studies was 0.96 (95% CI 0.91-1.03, *p* = 0.26; Figure 2). No significant heterogeneity was detected among the studies included for OS analysis (*I2* = 31%).

**Figure 3:**
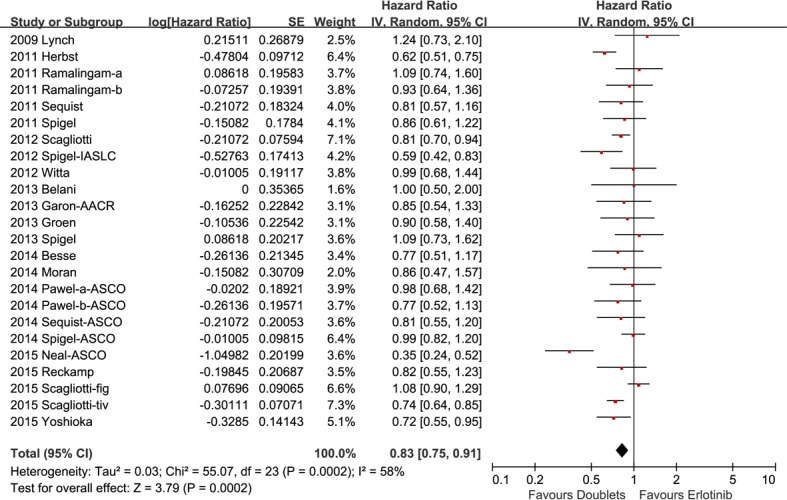
Forest plots for progression-free disease

The median PFS of the doublets group and single-agent group were 1.3 to 5.4 months and 1.5 to 3.5 months, respectively. Considering significant heterogeneity among the studies (*I2* = 58%), a random effect model was employed to estimate the pooled HR for PFS. Pooled PFS of patients treated with erlotinib plus the other targeted agent was superior to those treated with erlotinib alone (HR 0.83, 95% CI 0.75-0.91, *p* = 0.0002; Figure 3).

**Figure 4:**
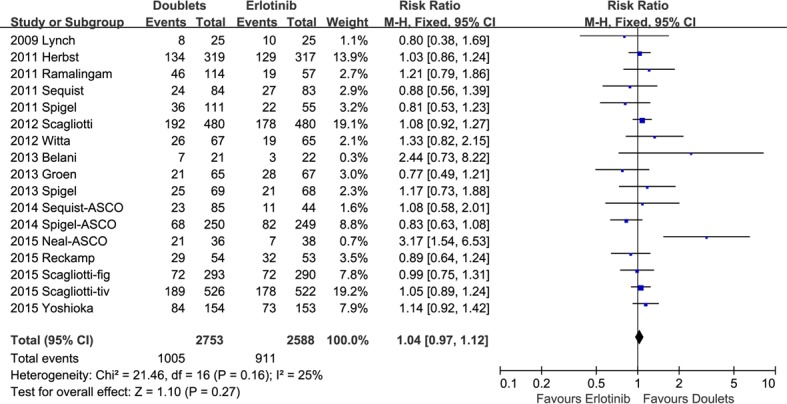
Forest plots for 1-year survival rate

**Figure 5:**
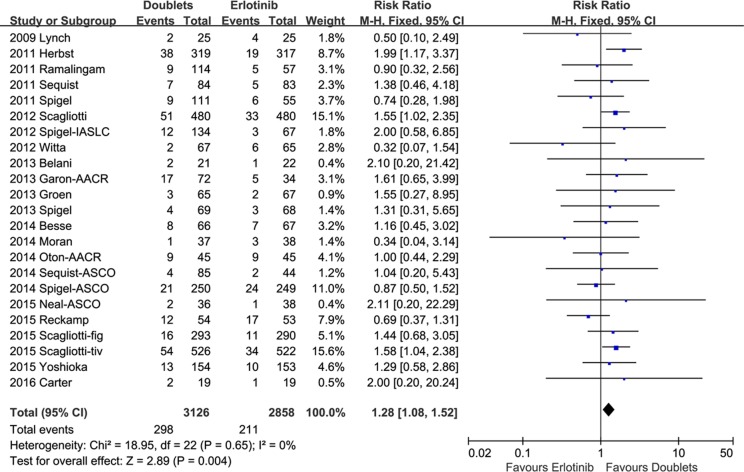
Forest plots for objective response rate

**Figure 6:**
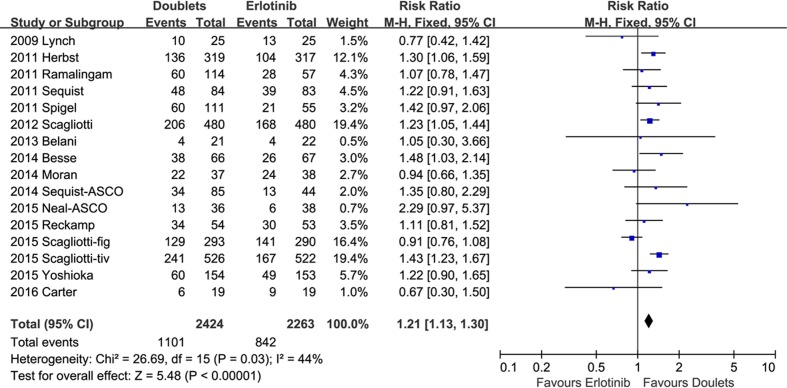
Forest plots for disease control rate

1-year SR did not significantly improve with doublets compared with single erlotinib (RR 1.04, 95% CI 0.97-1.12, *p* = 0.27; *I2* = 25%; Figure 4). However, ORR and DCR were in favor of the doublet targeted therapy (RR 1.28, 95 % CI 1.08-1.52, *p* = 0.004; *I2* = 0%; and RR 1.21, 95% CI 1.13-1.30, *p* < 0.00001; *I2* = 44%, respectively; Figures 5 and 6).

Neither phase II nor phase III trials subset analysis of OS revealed significant differences between the erlotinib-based combinations compared with the single agent (HR 0.91, 95 % CI 0.82-1.01, *p* = 0.08; *I2* = 34%; and HR 1.00, 95% CI 0.92-1.08, *p* = 0.92; *I2* = 16%, respectively; Table 3), whereas both phase II and phase III trials subgroup analysis showed improvement in PFS with doublets regimen over single erlotinb regimen (HR 0.83, 95 % CI 0.73-0.95, *p* = 0.007; *I2* = 45%; and HR 0.81, 95 % CI 0.69-0.96, *p* = 0.01 ; *I2* = 79%, respectively; Table 3).

**Table 3:** Sub-group analysis based on study characteristics

Sub-group	OS				PFS			
	*N*	HR (95%CI)	*p*	I-square, %	*N*	HR (95%CI)	*p*	I-square, %
**Phase**								
II	2035	0.91 (0.82, 1.01)	0.08	34	2035	0.83 (0.73, 0.95)	0.007	45
III	4033	1.00 (0.92, 1.08)	0.92	16	4033	0.81 (0.69, 0.96)	0.01	79
model	IV, fixed-effects model				IV, random-effects model			
**Mechanism**								
Anti-angiogenesis	2095	0.96 (0.86, 1.06)	0.42	0	2095	0.73 (0.62, 0.86)	0.0002	49
Anti-MET	2158	0.99 (0.86, 1.13)	0.86	24	2158	0.84 (0.72, 0.99)	0.03	54
Anti-angiogenesis & anti-MET	74	0.44 (0.29, 0.66)	<0.0001	NA*	74	0.35 (0.24, 0.52)	<0.00001	NA*
Anti-IGF-1R	829	0.98 (0.73, 1.30)	0.88	57	829	1.04 (0.90, 1.21)	0.55	0
Anti-ErbB3	341	1.12 (0.89, 1.43)	0.34	0	341	0.85 (0.68, 1.06)	0.16	0
Others	571	0.91 (0.74, 1.13)	0.4	0	571	0.91 (0.96, 1.09)	0.31	0
model	IV, random-effects model				IV, random-effects model			
***EGFR* status**								
Mutant	196	1.01 (0.32, 3.19)	0.98	65	105	1.09 (0.63, 1.88)	0.76	0
Wild-type	2589	0.89 (0.75, 1.06)	0.2	61	2205	0.68 (0.57, 0.83)	<0.0001	64
IHC-positive	297	1.10 (0.83, 1.46)	0.51	0	108	0.92 (0.58, 1.47)	0.73	0
IHC-negative	91	0.92 (0.56, 1.50)	0.74	NA*	31	0.95 (0.37, 2.47)	0.92	NA*
FISH-positive	105	1.34 (0.85, 2.12)	0.21	0	36	0.90 (0.41, 1.97)	0.79	NA*
FISH-negative	158	0.90 (0.47, 1.71)	0.74	52	102	0.87 (0.54, 1.41)	0.58	0
model	IV, random-effects model				IV, random-effects model			
***KRAS* status**								
Mutant	499	0.95 (0.76, 1.19)	0.64	34	102	0.23 (0.13, 0.41)	<0.00001	0
Wild-type	1530	0.93 (0.82, 1.05)	0.23	0	523	0.79 (0.64, 0.97)	0.03	12
model	IV, fixed-effects model				IV, fixed-effects model			

OS, overall survival; PFS, progression-free survival; HR, hazard ratio; CI, confidence interval; IV, inverse variance; I-square, inconsistency statistic; MET, mesenchymal-epithelial transition factor; IGF-1R, insulin-like growth factor-1 receptor; EGFR, epidermal growth factor receptor; IHC, immunohistochemistry; FISH, fluorescence in-situ hybridization;

*NA, not applicable, due to only one trail involved

Various targeted signaling pathways were involved in the 24 eligible studies. For a subgroup analysis, we divided different targets into six groups: anti-angiogenesis, anti-MET, anti-IGF-1R, anti-ErbB3 signaling, anti-angiogenesis plus anti-MET signaling and others. Overall, no significant differences existed in PFS or OS between combining targeted therapy and erlotinib monotherapy, except that patients treated with erlotinib plus anti-angiogenesis or anti-MET targeted agents showed improvement in PFS (HR 0.73, 95% CI 0.62-0.86, *p* = 0.0002; *I2* = 49%; and HR 0.84, 95% CI: 0.72-0.99, *p* = 0.03; *I2* = 54%, respectively) and the doulets erlotinib plus cabozantinib (anti-angiogenesis plus anti-MET signaling) group revealed significant improvement in both OS and PFS (HR 0.44, 95 % CI 0.29-0.66, *p* < 0.0001; and HR 0.35, 95 % CI 0.24-0.52, *p* < 0.00001, respectively; Supplementary Figures 1 and 2; Table 3).

11 studies provided the detailed analysis of OS in *EGFR* wild-type population. The pooled HR was 0.89 (95% CI 0.75-1.06, *p* = 0.2; *I2* = 61%; Supplementary Figure 3). Combining PFS of ten trials involving 2205 NSCLC harboring wild-type *EGFR* produced a significant improvement from the doublet targeted therapy (HR 0.68, 95% CI 0.57-0.83, *p* < 0.0001; *I2* = 64%; Supplementary Figure 4). Complete survival results of subgroup analysis based on *EGFR* gene mutations, protein expression and gene copy number were summarized in Table 3. No significant differences were observed expect for PFS in *EGFR* wild-type population mentioned above.

In patients with *KRAS* mutations, the pooled HR for OS and PFS for combination arm versus erlotinib arm were 0.95 (95% CI 0.76-1.19, *p* = 0.64; *I2* = 34%) and 0.23 (95% CI 0.13-0.41, *p* < 0.00001; *I2* = 0%), respectively. In *KRAS* wild-type population, the pooled HR for OS and PFS were 0.93 (95% CI 0.82-1.05, *p* = 0.23; *I2* = 0%) and 0.79 (95% CI 0.64-0.97, *p* = 0.03; *I2* = 12%), respectively (Supplementary Figure S5 and S6; Table 3).

### Publication bias

After assessment by Begg's test and Egger's test, no publication bias was found. The p values based on Begg's test for OS, PFS, ORR, DCR, 1-year SR in the total population were 0.941, 0.309, 0.712, 0.449, 0.387, respectively. For Egger's test, the p values were 0.768, 0.673, 0.166, 0.701, 0.521, respectively.

## DISCUSSION

EGFR inhibitors have been approved for the second-line treatment of advanced NSCLC, regardless of *EGFR* mutational status.[4] However, patients who initially benefit from EGFR-targeted therapy eventually develop resistance and have poor prolongation of survival. Currently, there are multiple trails combining molecular agents that target different signaling pathways, attempting to overcome drug resistance and optimize utilization of single-agent erlotinib.

Our meta-analysis focused on erlotinib-based doublets as subsequent treatment after disease progression with chemotherapy. We confirmed that combination therapy resulted in prolonged progression-free survival (PFS), better overall response rate (ORR) and disease control rate (DCR) as compared to erlotinib monotherapy, though similarities in overall survival and one-year survival rate were observed. Perhaps these results were not surprising because PFS, ORR and DCR were all tumor-based assessment end points, while OS analysis could be confounded by multiple factors such as cross-over, subsequent therapies and long post-progression survival. A recent study investigating trail-level associations between PFS, ORR and OS may supporting our viewpoint, which demonstrated a strong association between ORR and PFS, but no association existed between ORR and OS or between PFS and OS.[33]

Pan *et al.* had performed a meta-analysis about similar subjects based on published data updated in November 2012, which concluded that erlotinib-based doublets regimen significantly improved ORR and DCR compared with single erlotinib, but 1-year SR was not significantly improved for doublets.[34] Though these results were consistent with ours, only five studies involving 2,100 patients were included in the meta-analysis, while our study included 24 RCTs involving 6,196 patients. Furthermore, besides dichotomous data (ORR, DCR, 1-year SR), our study pooled the HR of time-to-event data (OS, PFS) as well, taking into account both the event and the timing of the event, to evaluate the efficacy of doublets therapy.

Qi *et al.* also conducted a meta-analysis evaluating combined targeted agents versus single-agent erlotinib, updated in May 2012. [35] The author included eight studies involving 2,417 patients and the efficacy endpoints were OS (HR 0.90, 95% CI 0.82-0.99, *p* = 0.024), PFS (HR 0.83, 95% CI 0.72-0.97, *p* = 0.018) and ORR (OR 1.35, 95% CI: 1.01-1.80, *p* = 0.04), all of which were in favor of the doublet targeted therapy according to the author's analysis. Whereas, our pooled data showed no statistical difference existed in OS between two arms. Possible explanation for this inconsistency was that another sixteen trails were incorporated and the number of participants was approximately 2.5-fold in our meta-analysis; Besides, the discordance might be associated with a three-arm trail investigating combing R1507 (given weekly or every 3 weeks) with erlotinib.[13] The trail reported HR for survival data with 90% confidence interval (CI), which should be transformed to 95% CI for further meta-analysis. For example, the 90% CI of HR for OS in ‘weekly’ group were 0.58-1.21 as reported yet it should be transformed to 95% CI, namely 0.54-1.30. Consequently, the revised pooled HR along with 95% CI for OS and PFS in the meta-analysis conducted by Qi *et al.* were 0.90 (95% CI 0.82-1.00, *p* = 0.04) and 0.82 (95%CI 0.71-0.95, *p* = 0.010). The revised *p* value (0.04) for pooled OS data, though statistically significant, was apparently larger than the author reported (0.024).

Subgroup analysis conducted by Qi *et al.* based on phases of trials, EGFR-status and KRAS status showed that there was just a tendency to improve PFS and OS in doublets, except that PFS for patients with *EGFR*-mutation or wild-type *KRAS* favored single agent. All of these subset results were not statistically significant. However, given that mutational status was rarely reported according the included trails in Qi's article, results must be interpreted with caution. Conversely, we performed similar subset analysis based on a relatively large number of patients and strict definitions of *EGFR* status, that is gene mutant or wild-type, IHC positive or negative and fluorescence in-situ hybridization (FISH) positive or negative. Significantly, PFS improvement in doublets in *EGFR* wild-type (*p* < 0.0001), *KRAS* mutant (*p* < 0.00001), *KRAS* wild-type (*p* = 0.03) was observed; While, PFS in *EGFR*-mutant patients showed a trend in favor of single-agent erlotinib (HR 1.09, 95%CI 0.63-1.88). The mechanism underlying these observations were unclear.

MET, a transmembrane tyrosine kinase receptor, is central to the processes of cancer cell migration, invasion, proliferation, and metastasis.[36] *MET* amplification and/or mutations are found in many human malignancies, including NSCLC, and predicts both resistane to EGFR TKIs and poor survival.[36–38] Thus, EGFR and MET may cooperate in driving tumorigenesis. Targeting angiogenesis is another promising strategy to improve survival in patients with many solid tumors, including NSCLC.[39]

Cabozantinib is a small molecule inhibitor of multiple receptor tyrosine kinases, including MET and vascular endothelial growth factor receptor 2 (VEGFR2). Notably, encouraging results of a randomized phase II trial testing cabozantinib, erlotinib or the combination in patients with *EGFR* wild-type NSCLC were presented during ASCO Annual Meeting 2015.[32] Cabozantinib, co-targeting angiogenesis and MET signaling plus erlotinib showed statistically significant improvement in both OS and PFS compared with erlotinib alone. Indeed, this trail was the only one of all included trials demonstrating overall survival benefits from combining therapy. Interestingly, our subset analysis based on different signaling pathways, involving 2,095 patients in anti-angiogenesis arm and 2,158 patients in anti-MET arm, suggested significant PFS improvement in patients treated with combined targeted agents including anti-angiogenesis (sorafenib, bevacizumab, pazopanib, sunitinib) and anti-MET (tivantinib, onartuzumab) targeted agents.

It should be noted that our analysis was limited to the use of individual patient data. All the outcome estimates were taken from published data, which tended to overestimate treatments effects. Furthermore, 10 of the 24 included RCTs were marked with “open-label” and the performance bias was assessed as “high risk”, which may decrease the quality of our meta-analysis.

Notably, according to *NCCN Guidelines Version 2.2017*, the standard of care in NSCLC now is to select patients based on their *EGFR* or *ALK* status. As for patients with EGFR mutation or ALK rearrangement, several targeted drugs are recommended as first line choose. Chemotherapy is an first option for *EGFR* or *ALK* negative patients. Therefore, RCTs studying erlotinib versus doublets targeted therapy are recommended being conducted in first-line setting. However, according to our update searching in PubMed database (*February 5, 2017*), there were only two articles reporting the efficacy of erlotinib compared to doublets in chemotherapy-native patients (no additional studies based in second-line therapy were found). One is an open-label randomized phase II study compared the combination of erlotinib and bevacizumab versus erlotinib alone in patients with non-squamous NSCLC harboring EGFR mutations in first-line setting.[40] The addition of bevacizumab to erlotinib conferred a significant improvement in PFS. Another investigating erlotinib plus Linsitinib (an IGF-1R inhibitor) or placebo in chemotherapy-naive patients. [41] Considering the limited number of relevant studies in first-line setting, our meta-analysis which seems lagging in the contemporary management of NSCLC is actually of great referential value in assessing efficacy of erlotinib versus doublets in first-line therapy. Future clinical studies should be designed based on the actual data in our meta-analysis.

From this analysis, we conclude that erlotinib combined with additional targeted agent, especially anti-angiogenesis and anti-MET agent, could provide superior clinical benefit to patients with previously treated advanced NSCLC. The efficacy of combination therapy for particular selected populations, such as *EGFR* wild-type population, need further investigation. The absence of a biomarker to identify sensitive populations is a major hurdle for optimal utilization.

## MATERIALS AND METHODS

### Protocol

This review was conducted and reported according to the Preferred Reporting Items for Systematic Reviews and Meta-Analysis (PRISMA) Statement issued in 2009. No ethical approval and patient consent are required as all analysis were based on previous published studies.

### Search strategy

A comprehensive and systematic search of the electronic databases (PubMed, Embase, and Cochrane) for studies published between inception and *February 2, 2016* was conducted. Applicable terms, such as “erlotinib”, “NSCLC”, “combin*” were used in the literature search with the filter “randomized control trial”. Relevant abstracts were searched and retrieved from American Society of Clinical Oncology (ASCO) databases.

### Study eligibility

Studies investigating combining molecular targeted therapy based on erlotinib versus erlotinib plus placebo or erlotinib alone in patients with advanced NSCLC (stage IV or IIIB) were eligible for inclusion. Studies that satisfied all the following criteria were included: (i) patients with histologically or cytologically confirmed stage IIIB or stage IV NSCLC and previously treated with at least one chemotherapy; (ii) assessing efficacy (and safety) profile of erlotinib-doublet targeted therapy versus single-agent erlotinib; (iii) phase II/III randomized controlled trials; (iv) at least one of the following outcome measures was extractable in an analyzable form: overall survival (OS), progression-free survival (PFS), objective response rate (ORR), disease control rate (DCR) or 1-year survival rate (SR).

The exclusion criteria were as follows: (i) duplicate reports; (ii) review articles; (iii) case reports; (iv) phase I and single-arm phase II trials owing to a lack of control groups; (v) ongoing studies; (vi) studies investigating targeted therapy as first-line treatment; (vii) studies not within the field of interest of this study.

### Data extraction

Data extraction from eligible studies were performed independently by two reviewers and disagreements were resolved by discussion and consensus with a third reviewer. The following information was extracted: the first author, year, trial phase, interventions, targeted pathways, number of subjects, median age, the percentage of female, smoking history, histology, ECOG performance status, stage, prior chemotherapy regimens, median OS, median PFS, ORR, DCR, 1-year SR, and the hazard ratio (HR) along with 95% confidence interval (CI) for the comparison of OS or PFS of erlotinib-based doublets-treated patients with that of patients receiving erlotinib alone. If the HR and 95% CI was not directly reported in the article, an estimation from the survival curve was made using Tierney's method.[42]

### Assessment of risk of bias in included studies

The methodological quality of RCTs was assessed using the risk of bias tool following the Cochrane Collaboration guidelines. Seven domains were employed for this part including random sequence generation, allocation concealment, blinding of participants, personnel or outcome assessment, incomplete outcome data, selective reporting and other sources of bias.

### Statistical analysis

The pooled HR for time-to-event outcomes (OS, PFS) and pooled relative risk (RR) for dichotomous data (ORR, DCR, and 1-year SR was calculated using the Review Manager 5.3 software statistical software. Heterogeneity assessed with the inconsistency statistic (*I2*) was interpreted as follows: *I2* = 0% indicates no heterogeneity, 0% < *I2* < 25% indicates the least heterogeneity, 25% ≤ *I2* < 50% indicates mild heterogeneity, 50% ≤ *I2* < 75% indicates moderate heterogeneity, and 75% ≤ *I2* indicates strong heterogeneity.[43] We employed a random-effects model in case of the existence of moderate or strong heterogeneity ( *I2* ≥ 50% ). Otherwise, a fixed-effects model was used. We pooled time-to event data using inverse variance method and dichotomous data with Mantel-Haenszel method. Subgroup analysis was performed according to phases of trials, targeted signaling pathways, *EGFR*-status and *KRAS*-status. *p* values < 0.05 were regarded as being statistically significant for all included studies. Publication bias was evaluated according to Begg's and Egger's test using the STATA 12.0 software statistical software.

## SUPPLEMENTARY MATERIALS FIGURES AND TABLES




