# Supplementary material for: Erlotinib-based doublet targeted therapy versus erlotinib alone in previously treated advanced non-small-cell lung cancer: a meta-analysis from 24 randomized controlled trials
Source: Oncotarget. 2017 May 31;8(42):73258–70. doi: 10.18632/oncotarget.18319 (PMC5641210; doi:10.18632/oncotarget.18319)
Supplement: Supplementary file 1 [file oncotarget-08-73258-s001.pdf]

Erlotinib-based doublet targeted therapy versus erlotinib alone in previously treated advanced non-small-cell lung cancer: a meta-analysis from 24 randomized controlled trials

SUPPLEMENTARY MATERIALS

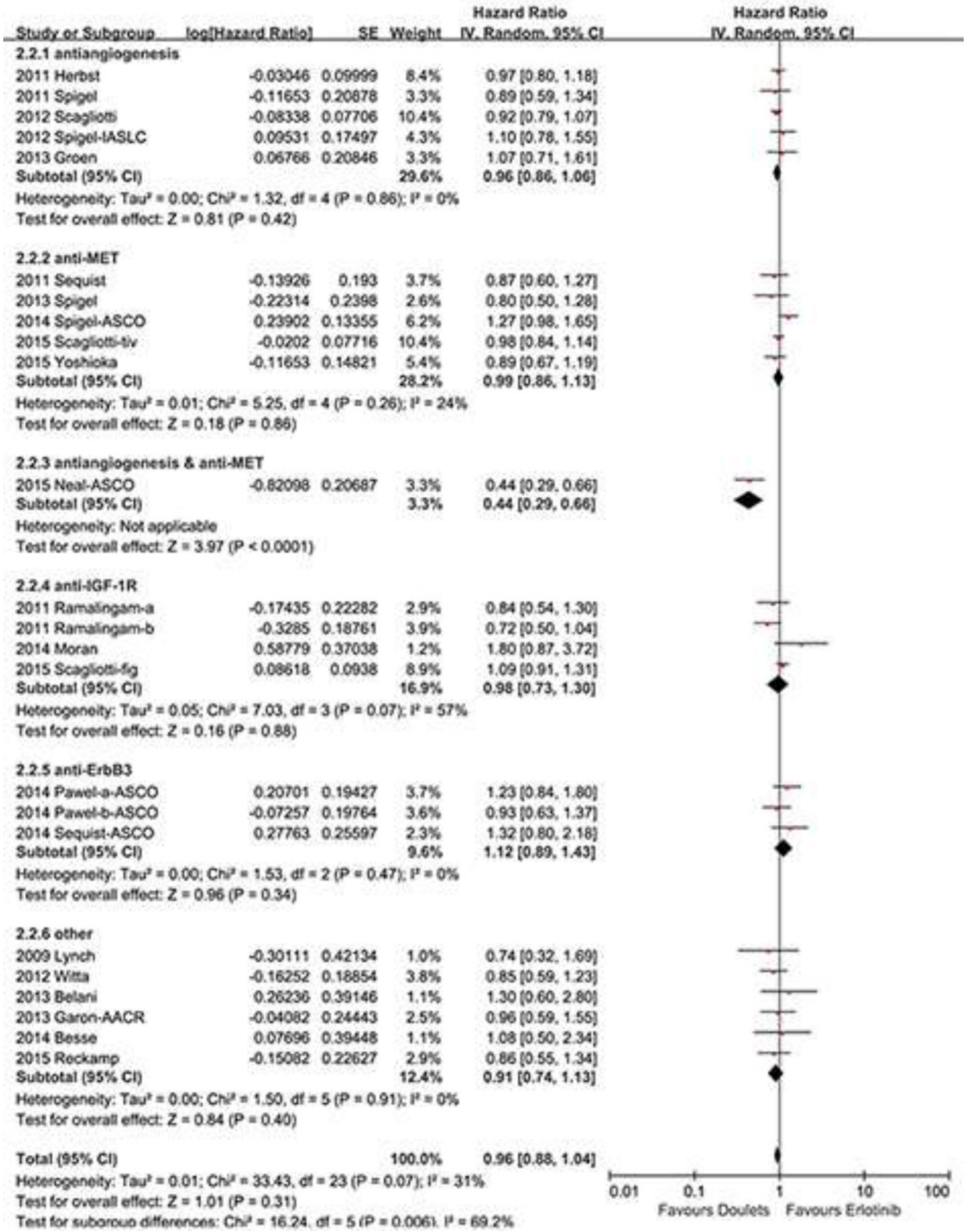

Supplementary Figure 1: Forest plots for overall survival according to targeted signaling pathways.

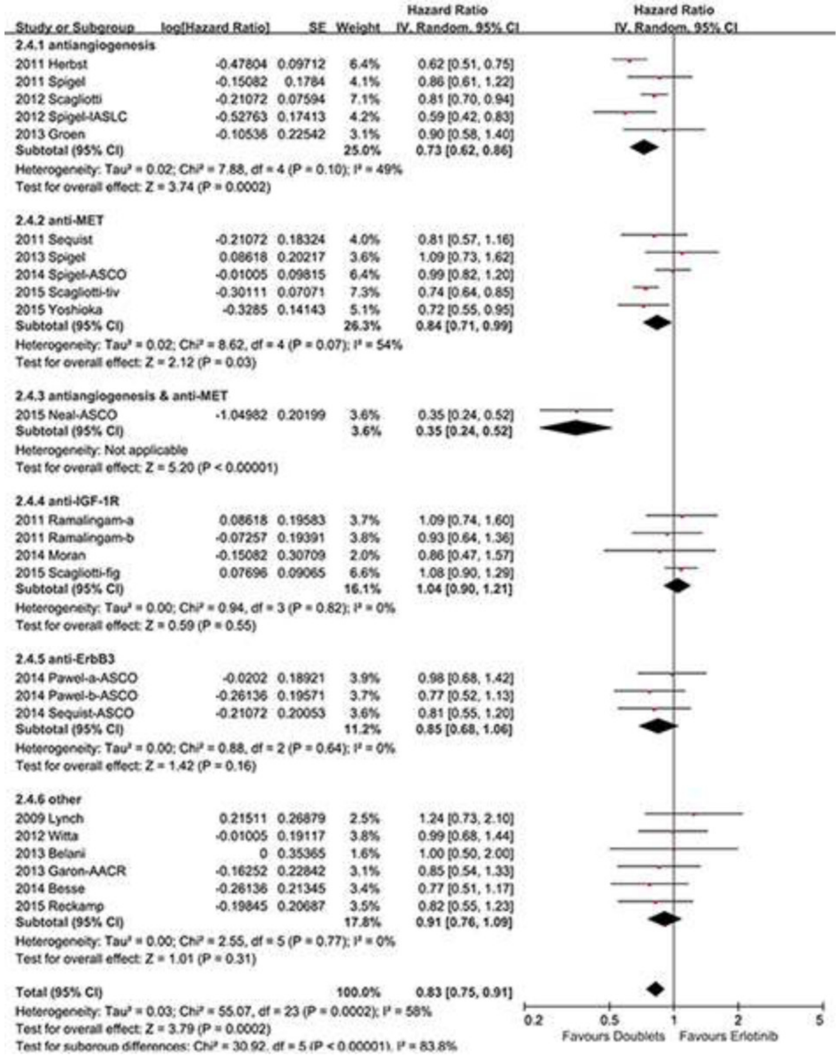

Supplementary Figure 2: Forest plots for progression-free disease according to targeted signaling pathways.

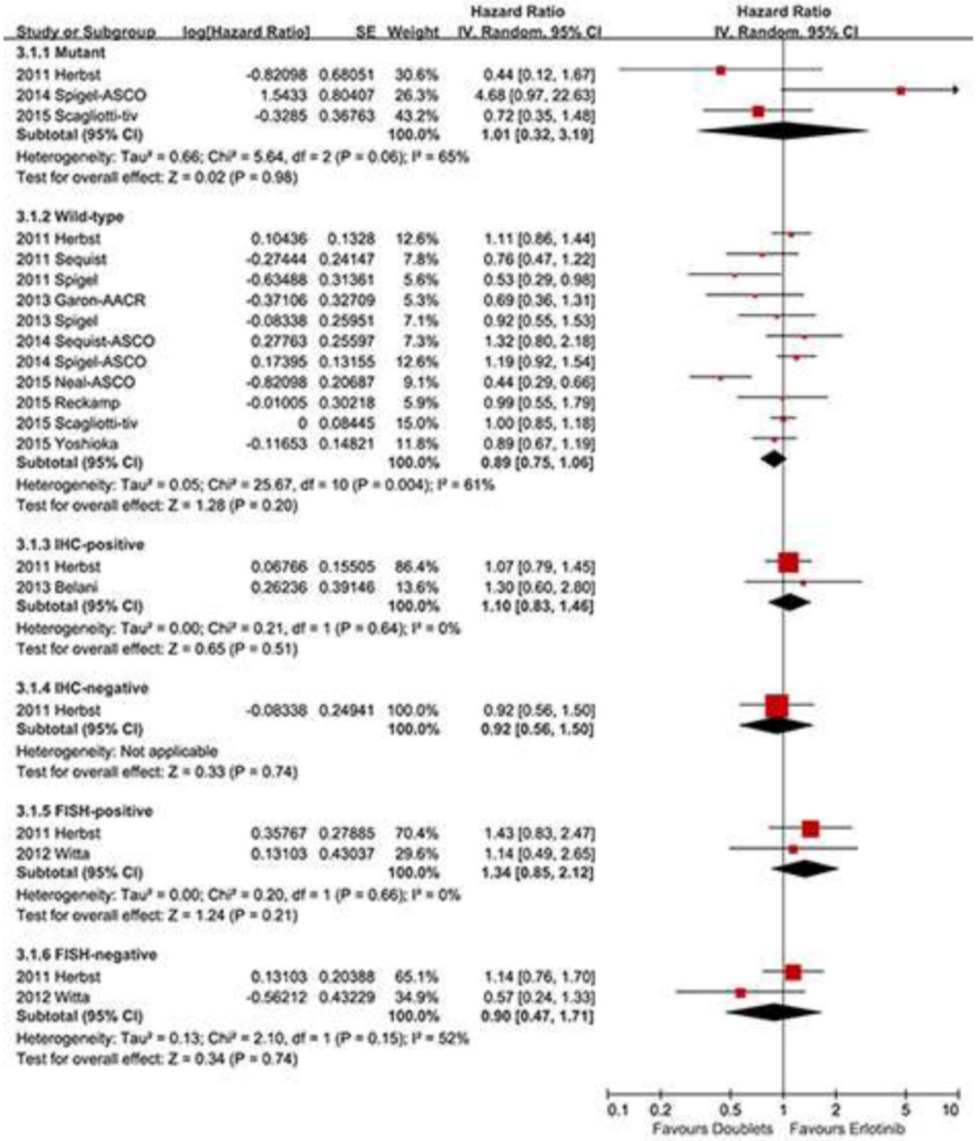

Supplementary Figure 3: Forest plots for overall survival according to *EGFR*-status. ***EGFR***, epidermal growth factor receptor.

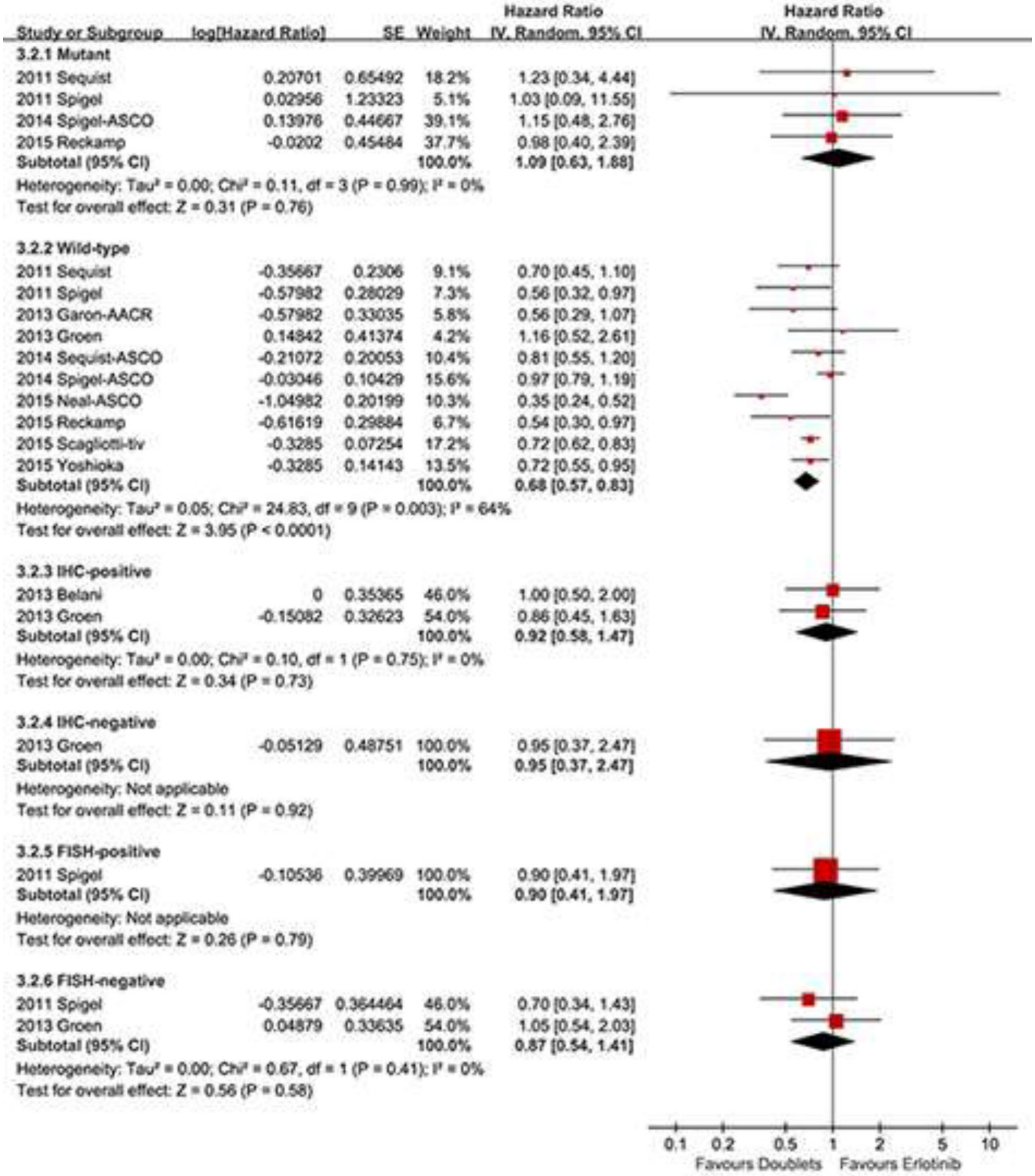

Supplementary Figure 4: Forest plots for progression-free disease according to *EGFR*-status. ***EGFR***, epidermal growth factor receptor.

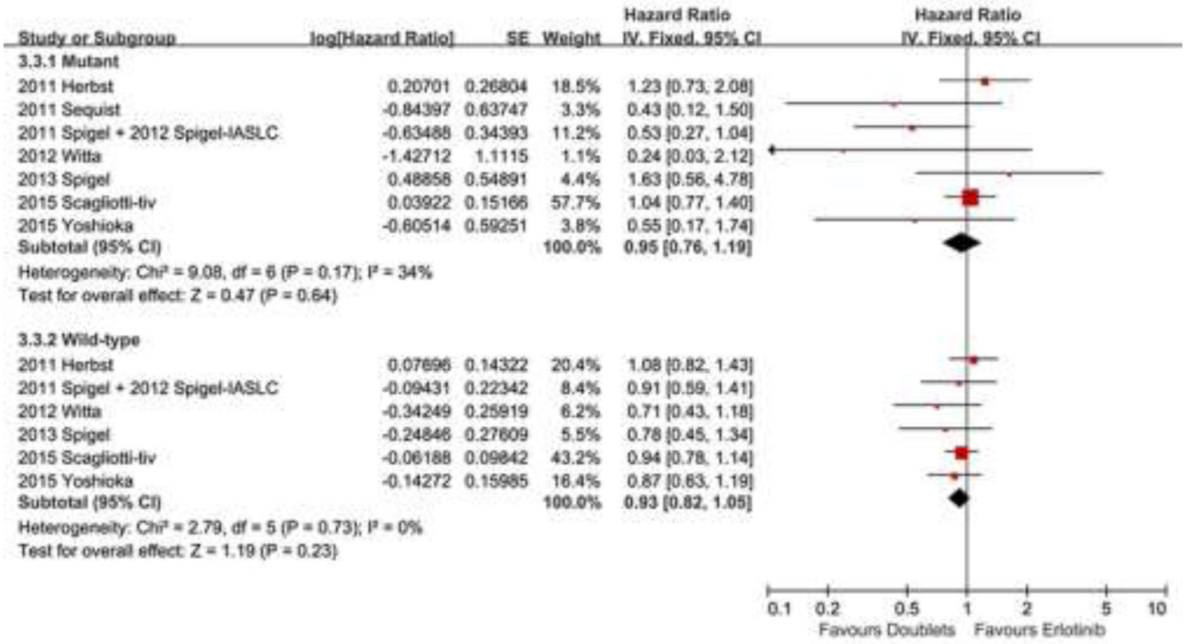

Supplementary Figure 5: Forest plots for overall survival according to *KRAS*-status.

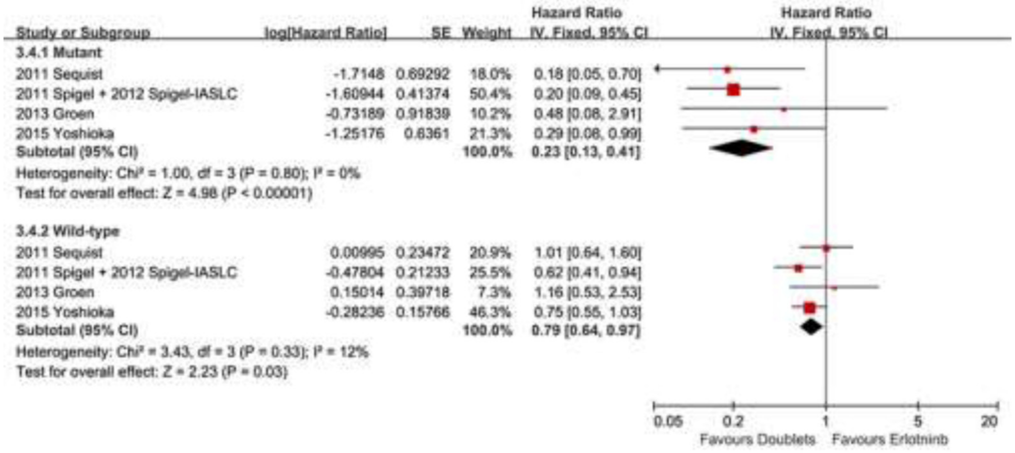

Supplementary Figure 6: Forest plots for progression-free disease according to *KRAS*-status.

For Supplementary Table see in Supplementary Files.
