# Supplementary material for: Erlotinib-based doublet targeted therapy versus erlotinib alone in previously treated advanced non-small-cell lung cancer: a meta-analysis from 24 randomized controlled trials
Source: Oncotarget. 2017 May 31;8(42):73258–70. doi: 10.18632/oncotarget.18319 (PMC5641210; doi:10.18632/oncotarget.18319)
Supplement: Supplementary file 2 [file oncotarget-08-73258-s002.doc]

| **supplementary Table S1.** Quality assessment of the randomized controlled trials based on the Cochrane risk of bias tool | | | | | | | | |
| --- | --- | --- | --- | --- | --- | --- | --- | --- |
| **Study** | **Year** | Random sequence generation | Allocation concealment | Blinding of partivipant and personnel | Blinding of outcome assessment | Incomplete outcome data | Selective reporting | Other bias |
|  |  | selection bias | selection bias | performance bias | detection bias | attrition bias | reporting bias |  |
| Lynch | 2009 | Uc | Uc | H | Uc | Uc | L | L |
| Herbst | 2011 | L | L | L | L | Uc | L | L |
| Ramalingam | 2011 | L | Uc | Uc | Uc | L | L | L |
| Sequist | 2011 | L | L | L | L | L | L | L |
| Spigel | 2011 | L | Uc | L | Uc | L | L | L |
| Scagliotti | 2012 | L | L | L | L | L | L | L |
| Spigel/IASLC | 2012 | L* | Uc | L | Uc | Uc | L | L |
| Witta | 2012 | L | Uc | L | Uc | L | L | L |
| Belani | 2013 | Uc | Uc | H | Uc | Uc | L | Uc |
| Garon/AACR | 2013 | Uc | Uc | H | Uc | Uc | L | L |
| Groen | 2013 | L | L | L | L | L | L | L |
| Spigel | 2013 | L | L | L | L | L | L | L |
| Besse | 2014 | Uc | Uc | H | Uc | L | L | L |
| Moran | 2014 | Uc | Uc | H | Uc | Uc | L | L |
| Oton/AACR | 2014 | Uc | Uc | H | Uc | Uc | L | L |
| Pawel/ASCO | 2014 | L* | L* | L | Uc | Uc | L | L |
| Sequist/ASCO | 2014 | L* | L* | H | Uc | Uc | L | L |
| Spigel/ASCO | 2014 | L* | L* | L | Uc | Uc | L | L |
| Neal/ASCO | 2015 | L* | Uc | H | Uc | Uc | L | L |
| Reckamp | 2015 | L | L | L | L | L | L | L |
| Scagliotti-fig | 2015 | L | L | H | Uc | L | L | Uc |
| Scagliotti-tiv | 2015 | L | L | L | L | L | L | L |
| Yoshioka | 2015 | L | L | L | L | L | L | L |
| Carter | 2016 | L | L | H | Uc | L | L | L |
| L, Low Risk; Uc, Unclear Risk; H, High Risk. | | | | | | | | |
| * reported "randomization" and we defined "selection bias" as "L", since earlier reports from the same investigators clearly describe use of random sequences and/or allocation concealment.. | | | | | | | | |
|  |  |  |  |  |  |  |  |  |
